# [WITHDRAWN] Activation of transsulfuration pathway to maintain cysteine is a thermogenic checkpoint for the conservation of energy

**DOI:** 10.21203/rs.3.rs-3069713/v1

**Published:** 2023-06-30

**Authors:** Aileen H. Lee, Yuki Sugiura, Yun-Hee Youm, Tamara Dlugos, Rae Maeda, Daniel Coman, Olga Spadaro, Sviatoslav Sidorov, Irina Shchukina, Sairam Andhey, Steven R. Smith, Eric Ravussin, Fahmeed Hyder, Maxim N. Artyomov, Vishwa Deep Dixit

**Affiliations:** 1 Department of Pathology; 2 Department of Comparative Medicine; 3 Department of Immunobiology, Yale School of Medicine, New Haven, CT 06520, USA.; 4 University of Kyoto, Japan; 5 Department of Radiology and Biomedical Imaging; 6 Department of Biomedical Engineering, School of Engineering and Applied Science, Yale University.; 7 Department of Pathology and Immunology Washington University School of Medicine, St. Louis, MO 63110, USA.; 8 Translational Research Institute for Metabolism and Diabetes, AdventHealth, Orlando, FL, USA.; 9 Pennington Biomedical Research Center, Baton Rouge, LA, USA.; 10 Yale Center for Research on Aging, Yale School of Medicine, New Haven, CT 06520, USA

## Abstract

The authors have requested that this preprint be removed from Research Square.

